# Mucinous adenocarcinoma in kidneys with developmental anomalies - a report of two cases

**DOI:** 10.1186/s12894-024-01637-y

**Published:** 2024-11-15

**Authors:** Kasi Viswanath Gali, Arun Chawla, K. R. Surag, Sunil Pillai Bhaskara, Padmaraj Hegde

**Affiliations:** Department of Urology, Kasturba Medical College, Manipal Academy of Higher Education, Manipal, Karnataka 576104 India

**Keywords:** Mucinous tumours, Cystadenocarcinoma, Renal anomalies, Horse-shoe Kidney, Duplex Moiety, Renal adenocarcinoma, Renal Mucinous tumour

## Abstract

**Background:**

Primary mucinous adenocarcinomas of the kidney are rare and pose a challenge for preoperative diagnosis. The histogenesis of these tumours remains largely unknown, with three proposed theories: chronic irritation, differentiation of celomic epithelium, and kidney maldevelopment. Here, we present two cases of renal mucinous adenocarcinoma in patients with developmental renal anomalies, specifically a duplex collecting system and a horseshoe kidney.

**Case Presentation:**

First, A 50-year-old male presented with loin pain and jelly-like discharge in urine with a duplex collecting system and gross hydronephrosis of the upper moiety on imaging. The patient underwent upper polar nephrectomy with controlled drainage of 1.5 L of mucinous fluid. Histopathology was suggestive of mucinous borderline cystic neoplasm with invasive microcarcinoma. The patient presented one year later, with a hydronephrotic lower moiety of the left kidney and a rectus abdominis mass. Fine needle aspiration biopsy of the mass revealed papillary adenocarcinoma with histological resemblance to the renal pelvis lesion, establishing it as a metastasis from the primary renal malignancy. Second, A 53-year-old male who had undergone right laparoscopic cyst deroofing for a symptomatic renal cyst, whose postoperative histology revealed findings consistent with mucinous adenocarcinoma, presented with flank pain and palpable retroperitoneal mass. Imaging revealed a horseshoe kidney morphology with a large multilobulated hypodense non-enhancing cystic lesion arising from the right kidney. Cyst excision with right open radical nephrectomy was performed. Gross examination revealed multiple cystic spaces replacing renal parenchyma, filled with gelatinous material. Microscopy was suggestive of recurrent mucinous adenocarcinoma.

**Conclusions:**

Renal mucinous cystadenocarcinomas can be associated with anomalous kidneys. Definitive diagnosis relies on histopathology, and these tumours are recognized for their aggressive nature. Complete resection is the preferred treatment, but further studies are needed to assess the efficacy of adjuvant treatment, given the poor prognosis and high likelihood of recurrence.

**Clinical trial number:**

Not applicable.

## Background

Primary mucinous adenocarcinoma of the renal pelvis, first reported in 1960 [1], remains exceptionally rare, with fewer than 100 documented cases. Preoperative diagnosis is challenging, often mimicking renal cystic disease [2, 3]. Histogenesis of primary renal mucinous cystadenocarcinoma remains largely unknown, with three proposed theories: chronic irritation, differentiation of celomic epithelium, and kidney maldevelopment. We present two cases of renal mucinous cystadenocarcinoma in patients with kidney developmental anomalies. Our study aims to add these rare cases to the limited existing literature, helping to improve understanding of this clinical condition.

## Case report

### Case 1

**Fig. 1:**
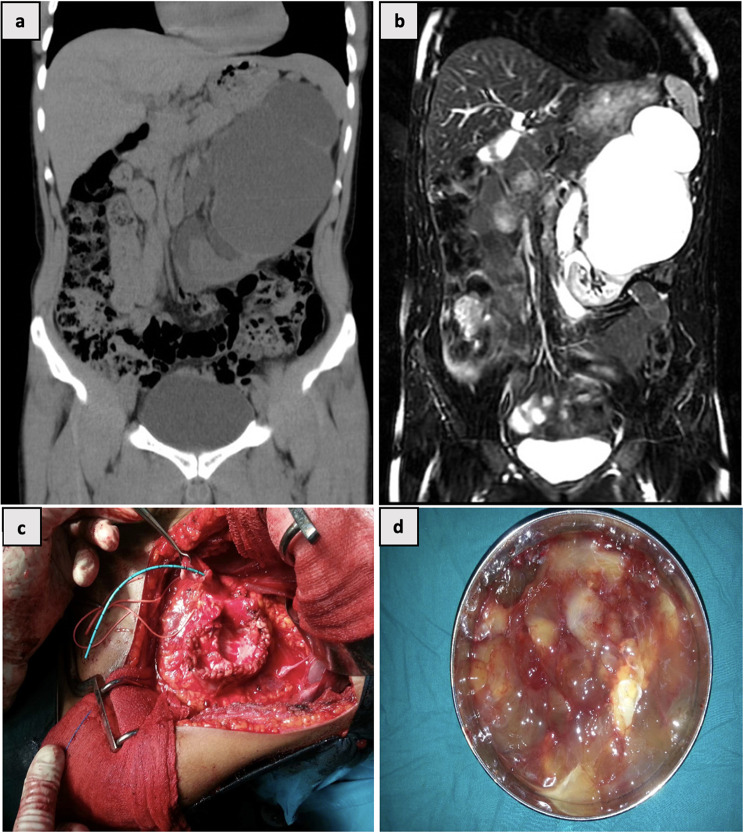
**(a)** Plain CT KUB showing a duplex left collecting system with gross hydronephrosis of left upper moiety **(b)** MR Urogram T2 images showing duplication of collecting system on left side with two ureters noted in proximal & middle 1/3rd and gross dilatation of upper moiety PCS and ureter **(c)** Intra op image of upper moiety nephrectomy (d) Thick Mucus drained from upper moiety

A 50-year-old male presented with left loin pain, jelly-like discharge in urine, and elevated serum creatinine (1.9 mg/dl). Non-contrast CT revealed a duplex collecting system with gross hydronephrosis of the left upper moiety (Fig. 1a). MR Urogram-T2 images demonstrated an incomplete duplex collecting system with gross hydronephrosis of left upper moiety with no appreciable parenchyma (Fig. 1b). Left retrograde pyelogram revealed a grossly distended upper moiety and ureteral confluence at L3 level. The patient underwent upper polar nephrectomy (Fig. 1c) with controlled drainage of 1.5 L of mucinous fluid (Fig. 1d) without any gross spillage. Postoperatively, the patient had an uneventful recovery and was discharged on the third postoperative day.

**Fig. 2:**
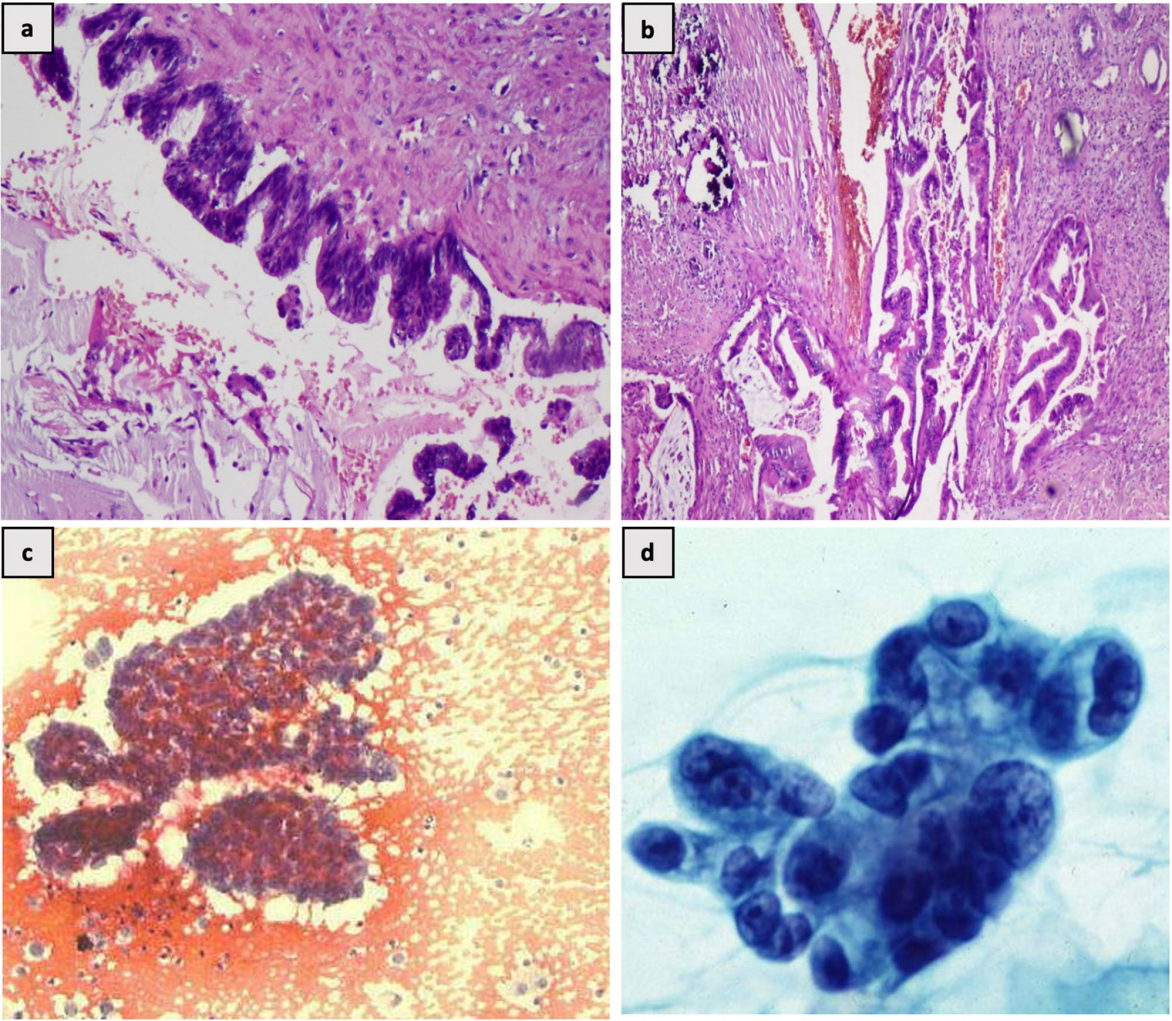
**(a)** micropapillary projections lined by mucinous epithelium and luminal mucin. H & E x 100 **(b)** focus of microcarcinoma showing minimal invasion of underlying stroma. H & E x 40 **(c)** Fine needle aspirate from abdominal swelling shows papillary fragments of malignant cells, suggestive of papillary adenocarcinoma PAP stain, 200X **(d)** malignant cells with pleomorphic nuclei, coarse chromatin and mucinous cytoplasm, PAP stain, 400X

Gross examination revealed a dilated pelvicalyceal system filled with mucin, thinning parenchyma, and a few tiny papillary projections. Histopathological analysis identified pseudostratified tall columnar epithelium with focal microcarcinoma invasion (Fig. 2a and b). Immunohistochemistry showed diffuse CK7, CK20 positivity, and focal CDX2 positivity, leading to a diagnosis of mucinous borderline cystic neoplasm with invasive microcarcinoma.

After being lost to follow up, the patient presented one year later, with a hydronephrotic lower moiety of the left kidney and a lower abdominal mass. MR imaging identified a mass in the rectus abdominis muscle. Fine needle aspiration biopsy (Fig. 2c and d) revealed clusters and syncytial papillae composed of malignant cells having increased N: C ratio, pleomorphic nuclei and coarse chromatic in the background of haemorrhage, proteinaceous material and mucin, suggestive of papillary adenocarcinoma with histological resemblance to the renal pelvis lesion, establishing it as a metastasis from the primary renal malignancy.

## Case 2

**Fig. 3:**
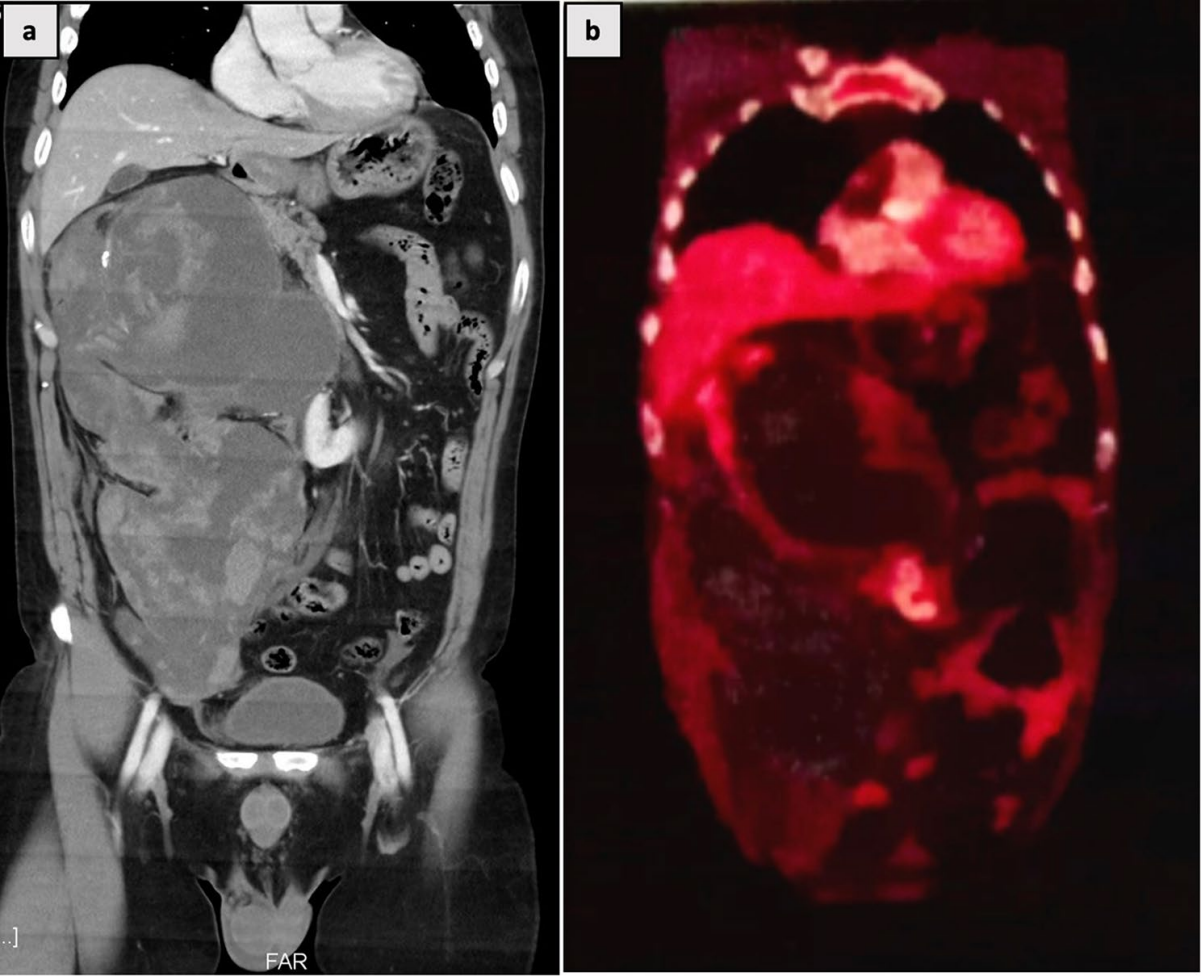
**(a)** horseshoe kidney morphology with a well-defined multilobulated hypodense non-enhancing cystic lesion in the right hypochondrium extending to the right lumbar region **(b)** FDG-PET showing an FDG avid soft tissue density thickening noted in the region of the right kidney with a non-FDG avid multiloculated hypodense non-enhancing collections with irregular rim calcifications

A 53-year-old male presented with right flank pain and a palpable retroperitoneal mass. The patient had undergone right laparoscopic cyst deroofing for a renal cyst at another medical facility approximately 6 months prior. The postoperative histology revealed findings consistent with mucinous adenocarcinoma. Contrast enhanced CT of the abdomen depicted a horseshoe kidney morphology with a well-defined multilobulated hypodense non-enhancing cystic lesion in the right hypochondrium extending to the right lumbar region, measuring 15.3 × 12.8 × 15.9 cm, with a thin rim of right renal parenchyma noted along its supero-lateral aspect (Fig. 3a). FDG PET scan confirmed non-FDG avid multiloculated hypodense non-enhancing collections with irregular rim calcifications. An anterolaterally FDG avid soft tissue density thickening measuring approximately 1.5 × 4.5 × 6.6 cm was noted in the region of the right kidney (Fig. 3b).

**Fig. 4:**
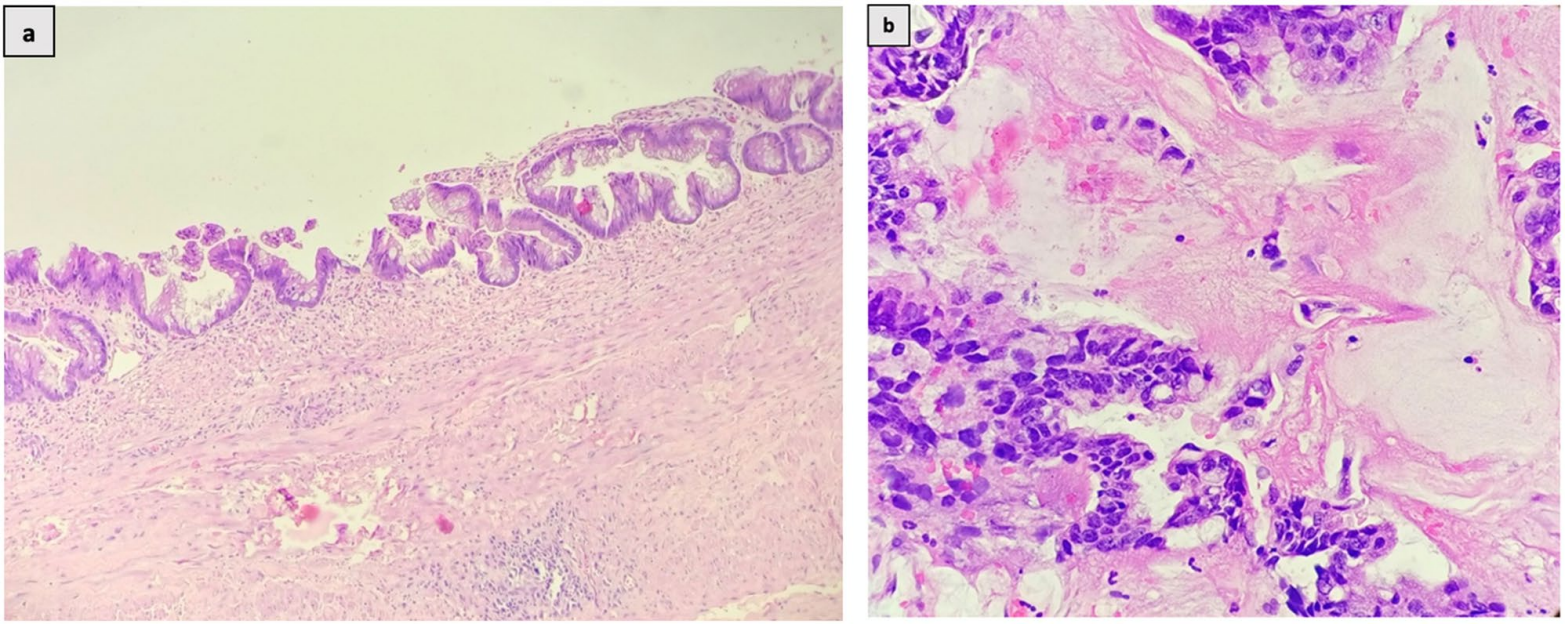
**(a)** Malignant mucinous epithelium lining the cyst wall (H&E; X100) **(b)** Malignant mucinous glands seen infiltrating the stroma with extravasated pools of mucin

Cyst excision with right open radical nephrectomy was performed, during which the cyst wall opened up, and gelatinous thick mucus with clots was aspirated with minimal spillage. Gross examination revealed multiple cystic spaces replacing renal parenchyma, filled with gelatinous material. Microscopy (Fig. 4a and b) revealed cyst walls lined by epithelium showing pleomorphic, hyperchromatic nuclei with stratification, vesicular chromatin, prominent nucleoli, and mucinous cytoplasm. Pools of extravasated mucin were observed, suggestive of recurrent mucinous adenocarcinoma. The postoperative period was uneventful, and the patient was discharged on the fourth postoperative day. Follow-up at 6 months was unremarkable.

## Discussion

Mucinous adenocarcinoma is a rare occurrence on the kidney, commonly arising from the renal pelvis but can also occur within the renal parenchyma [4]. It can be primary or secondary, depending on the tumour’s origin. It is crucial to rule out primary tumours from the pancreas, ovary, and appendix, particularly in the context of pseudomyxoma peritonei [5]. Limited evidence exists for secondary renal mucinous cystadenocarcinoma, with a single reported case of appendicular cystadenocarcinoma presenting as a renal tumor [6]. Both of our cases presented as primary renal mucinous adenocarcinomas, as there were no tumours observed in other organs.

The histogenesis is uncertain. The theory of chronic irritation, often associated with obstructing urinary calculi and recurrent infections, is a commonly proposed explanation in reported cases [7, 8]. Another hypothesis involves the differentiation of celomic epithelium, where mesothelium undergoes mucinous metaplasia [7, 9].

Less commonly, renal mucinous cystadenocarcinoma is found in patients with developmental renal anomalies, supporting the theory of renal maldevelopment [10–12]. Our Case 1 represents, to the best of our knowledge, the first documented instance of a duplex collecting system presenting with muconephrosis. It is postulated that sequestration of segment of renal pelvic epithelium within the renal parenchyma during maldevelopment predisposes to the development of these tumours [12]. This explains the presentation in our case 2 who presented as a renal parenchymal cyst. Presently, the association of Renal Mucinous adenocarcinoma and Developmental Renal anomalies remains hypothetical, and further studies focusing on molecular or immunohistochemical characteristics are needed to explore and potentially confirm this link.

Clinical presentation ranges from asymptomatic renal cysts to those presenting with pseudomyxoma peritonei. Mucosuria is a distinctive feature but is seldom observed [13]. Factors raising suspicion include prolonged symptom duration, association with calculi, presence of hydronephrosis, and a preoperative appearance suggestive of an inflammatory condition [14]. Our Case 1 showed mucosuria, indicating potential mucinous tumours. Conversely, Case 2, initially diagnosed as a benign renal cyst, lacked suggestive clinical history.

There are no specific diagnostic characteristics for mucinous cystadenocarcinoma on cross-sectional imaging. Laboratory testing is nonspecific. In some reported cases, primary mucinous adenocarcinomas of the renal pelvis have been linked to elevated levels of CEA or CA19-9^15^. Both our cases did not have any suggestive radiological or laboratory findings. The definitive diagnosis of mucinous cystadenocarcinoma is established through histopathology [16]. Immunohistochemistry studies show positive staining for CDX2, MUC2, and CK20, CK 7, EMA, and CEA [17]. This aligns with our findings in Case 1, which exhibited diffuse CK7, CK20 positivity, and focal CDX2. However, immunohistochemistry was not performed in Case 2 due to financial constraints.

Treatment options range from partial to radical nephrectomy [9, 16]. Some recommendations also include additional ureterectomy with a bladder cuff, similar to the approach for upper tract urothelial carcinoma (UTUC) [9, 10, 16]. Some authors have explored the role of adjuvant chemotherapy and radiotherapy [15]. However, the impact on prognosis remains uncertain. In Case 1 the patient experienced tumor recurrence in the lower moiety with uncertainty about it being metachronous or resulting from tumor implantation from spillover of contents during the initial procedure possibly explaining the metastatic lesion in the rectus abdominis. In Case 2 tumor recurrence post cyst deroofing suggests tumor cell implantation from the cyst fluid spillover. Therefore in any surgical intervention involving a cystic renal lesion efforts must be made to avoid cyst fluid spillage to reduce the risk of recurrence. This also highlights the potential need for adjuvant therapy to prevent tumour recurrence.

Generally, the prognosis appears to be poor, with overall survival of 2–5 years [9]. Recent literature suggests a disease-free follow-up of 28 months [18]. However, both of our cases exhibited recurrence within 12 months, underscoring the aggressive nature of these tumours.

## Conclusions

Renal mucinous cystadenocarcinomas can be associated with anomalous kidneys. Definitive diagnosis relies on histopathology, and these tumours are recognized for their aggressive nature. Complete resection is the preferred treatment, but further studies are needed to assess the efficacy of adjuvant treatment, given the poor prognosis and high likelihood of recurrence.

## Data Availability

The datasets used and/or analyzed during the current study are available from the corresponding author on reasonable request.

## Abbreviations

CT: Computed Tomography
MR: Magnetic Resonance Imaging
FDG PET: Fluorodeoxyglucose-Positron Emission Tomography
